# NR4A1 Regulates Tamoxifen Resistance by Suppressing ERK Signaling in ER-Positive Breast Cancer

**DOI:** 10.3390/cells10071633

**Published:** 2021-06-29

**Authors:** Yu Cheon Kim, Clara Yuri Kim, Ji Hoon Oh, Myoung Hee Kim

**Affiliations:** 1 Embryology Laboratory, Department of Anatomy, College of Medicine, Yonsei University, Seoul 03722, Korea; mdds12@yuhs.ac (Y.C.K.); ck1320@nyu.edu (C.Y.K.); 2 Brain Korea 21 Project for Medical Science, College of Medicine, Yonsei University, Seoul 03722, Korea

**Keywords:** NR4A1, ERK signaling pathway, tamoxifen resistance, breast cancer

## Abstract

Endocrine therapy is used to treat estrogen receptor (ER)-positive breast cancer. Tamoxifen is effective against this cancer subtype. Nonetheless, approximately 30% of patients treated with tamoxifen acquire resistance, resulting in therapeutic challenges. NR4A1 plays key roles in processes associated with carcinogenesis, apoptosis, DNA repair, proliferation, and inflammation. However, the role of NR4A1 in tamoxifen-resistant ER-positive breast cancer has not yet been elucidated. Here, we propose that NR4A1 is a promising target to overcome tamoxifen resistance. NR4A1 gene expression was downregulated in tamoxifen-resistant MCF7 (TamR) cells compared to that in MCF7 cells. Kaplan-Meier plots were used to identify high NR4A1 expression correlated with increased survival rates in patients with ER-positive breast cancer following tamoxifen treatment. Gain and loss of function experiments showed that NR4A1 restores sensitivity to tamoxifen by regulating cell proliferation, migration, invasion, and apoptosis. NR4A1 localized to the cytoplasm enhanced the expression of apoptotic factors. In silico and in vitro analyses revealed that NR4A1 enhanced responsiveness to tamoxifen by suppressing ERK signaling in ER-positive breast cancer, suggesting that the NR4A1/ERK signaling axis modulates tamoxifen resistance. These results indicate that NR4A1 could be a potential therapeutic target to overcome tamoxifen resistance in ER-positive breast cancer.

## 1. Introduction

Breast cancer is one of the most common cancers in women worldwide [1], and it can be classified into four molecular subtypes based on gene expression profiling [2]. Among these subtypes, estrogen receptor (ER)-positive breast cancer accounts for approximately 70% of all breast cancers and are generally treated with endocrine therapies, such as tamoxifen and aromatase inhibitor [3]. Tamoxifen, which is the most widely used endocrine therapy, has significant therapeutic effects; however, approximately one-third of patients with ER-positive breast cancer treated with tamoxifen for 5 years acquire resistance to the drug, which reduces its therapeutic effect and shortens the survival of patients [4,5,6]. However, the molecular targets for predicting tamoxifen resistance are still insufficient. Therefore, discovering molecular mechanisms and genes associated with tamoxifen resistance is required to develop novel therapeutic strategies to overcome tamoxifen resistance in patients with ER-positive breast cancer.

The NR4A subfamily, which consists of NR4A1 (Nur77), NR4A2 (Nurr1), and NR4A3 (Nor1), belongs to the orphan nuclear receptor family with members whose ligands are unknown, which function as transcription factors [7]. NR4A subfamily members are early response genes affected by various signals in a variety of cells and tissues and are known to be involved in cellular activities such as proliferation, migration, apoptosis, DNA repair, inflammation, metabolism, and angiogenesis [8,9]. These functions depend on cellular localization and post-translational modification as well as gene expression [10]. Among NR4A subfamily members, the function of NR4A1 is the most known, and NR4A1 acts as an oncogene or a tumor suppressor in diverse cancer models, such as colon cancer, liver cancer, lung cancer, melanoma, and acute myeloid leukemia [11,12,13,14,15]. Especially, the loss of NR4A1 and NR4A3 in mice leads to the rapid development of lethal acute myeloid leukemia, indicating tumor suppressor-like activity for these receptors [15,16,17]. Similarly, the role of NR4A1 in breast cancer has been controversial. Recent studies have shown that ectopic expression of NR4A1 promotes invasion and metastasis by activating TGF-β/SMAD signaling in breast cancer [18]. Further, the translocation of NR4A1 from the nucleus to the cytoplasm has been found to induce apoptosis in all-trans retinoic acid-induced breast cancer cells [19]. NR4A1 also inhibits growth and metastasis in triple-negative breast cancer [20]. However, the functional role and associated molecular mechanism of NR4A1 in tamoxifen-resistant breast cancer have not been elucidated.

The mitogen-activated protein kinase (MAPK) cascade, including extracellular signal-regulated kinase (ERK), c-Jun NH_2_-terminal kinase, and p38, is a signaling pathway that regulates various cellular functions [21] and is activated in most cancer models [22]. In particular, several studies have suggested that the ERK signaling pathway plays important roles in breast cancers [23]. Activation of ERK signaling in breast cancer is not only associated with augmented tumor growth and metastasis [24,25] but also with tamoxifen resistance [26]. Activated ERK signaling reduces the response and effectiveness of tamoxifen treatment and causes poor prognosis in patients with ER-positive breast cancer [27,28]. Therefore, the inhibition of ERK signaling associated with tamoxifen resistance improves the survival rate of patients with ER-positive breast cancer because of their increased response to tamoxifen therapy.

In this study, we explored the role and regulatory mechanism of NR4A1 in tamoxifen-resistant ER-positive breast cancer cells and patient sample data retrieved from a public database cBioportal. We found that NR4A1 increases responsiveness to tamoxifen, resulting in reduced cell growth, invasion, and migration, and induced apoptosis following tamoxifen treatment. NR4A1, located in the cytoplasm, also enhances the expression of apoptosis-related molecules. Mechanistically, NR4A1 re-sensitizes the response to tamoxifen by suppressing the ERK signaling pathway. Our results demonstrate that NR4A1 reduces tamoxifen resistance by regulating ERK signaling in ER-positive breast cancer, suggesting that it could be a novel diagnostic marker and/or therapeutic target to overcome tamoxifen resistance.

## 2. Materials and Methods

### 2.1. Cell Culture and Treatment

MCF7, T47D, tamoxifen-resistant MCF7 (TamR), and T47D (T47D-TamR) breast cancer cells were used in this study. MCF7 cells were provided by Kyung Tae Kim (National Cancer Center, Korea). TamR cells were established as an in vitro model for tamoxifen resistance by treating MCF7 cells with 1 μM 4-hydroxytamoxifen (Sigma, MO, USA) for at least 6 months [29]. T47D and T47D-TamR cells were provided by Dr. Mi-Ock Lee (Seoul National University, Korea). MCF7 and TamR cells were cultured in Dulbecco’s modified Eagle’s medium (WelGENE Inc., Daegu, Korea) supplemented with 10% fetal bovine serum (WelGENE Inc., Daegu, Korea) and 1% penicillin-streptomycin (WelGENE Inc., Daegu, Korea). T47D and T47D-TamR cells were cultured in RPMI 1640 (WelGENE Inc., Daegu, Korea) with the same supplementation. TamR cells were cultured in a medium containing 1 μM 4-hydroxytamoxifen (Sigma, MO, USA). Cells were grown at 37 °C in a 5% CO_2_ incubator. For some experiments, NR4A1-knockdown cells were treated with 50 μM ERK1/2 inhibitor U0126 (Calbiochem, CA, USA) for 2 h prior to analysis.

### 2.2. Plasmid and siRNA Transfection

For the overexpression study, the full-length coding sequence of the NR4A1 gene was cloned into a pcDNA3-HA-tagged vector, and 7.5 × 10^5^ cells were transfected with pcDNA3-NR4A1 plasmid for 24 h using Attractene (Qiagen, Hilden, Germany) according to the manufacturer’s protocol. To generate stable cell lines, pcDNA3-NR4A1 plasmid-transfected cells were treated with 500 μg/mL G418 for 2 weeks. For the knockdown study, 3.5 × 10^5^ cells were transfected with 50 nM of NR4A1-siRNA (Genolution, Seoul, Korea) or control siRNA (Genolution, Seoul, Korea) for 48 h using G-fectin (Genolution, Seoul, Korea) following the manufacturer’s protocol. siRNAs were designed and purchased from Genolution. The sequence of siRNA was as follows: siNR4A1 #1: Sense—5′-GCUACACAGGAGAGUUUGAUU-3′, Antisense—5′-UCAAACUCUCCUGUGUAGCUU-3′; siNR4A1 #2: Sense—5′-GAAGUUGUCCGAACAGACAUU-3′, Antisense— 5′-UGUCUGUUCGGACAACUUCUU-3′.

### 2.3. Total RNA Isolation and Quantitative Real-Time PCR

Total RNA was extracted from cells using TRIzol reagent (Invitrogen, CA, USA) following the manufacturer’s instructions. cDNA was synthesized from 1 μg of total RNA by reverse transcription using ImProm-II Reverse Transcriptase (Promega, WI, USA). PCR was performed using Taq DNA polymerase (Bioneer, Seongnam, Korea) with the following conditions: initial denaturation for 5 min at 95 °C, followed by 28–36 cycles at 95 °C for 40 s, at 56 °C for 20 s (depending on target genes), and at 72 °C for 30 s. For quantitative real-time PCR, Power SYBR Green PCR Master Mix (Applied Biosystems, CA, USA) and StepOnePlus^TM^ Real-Time PCR System (Applied Biosystems, San Francisco, CA, USA) were used. All PCR reactions were performed at least in triplicate, and gene expression was normalized relative to that of human β-Actin and GAPDH, which were used as internal controls. Primers used for PCR were as follows: Human NR4A1: Forward—5′-CCAAGTACATCTGCCTGGCTA-3′, Reverse—5′-GACAACTTCCTTCACCATGCC-3′; Human NR4A2: Forward—5′- CCCAGTGGAGGGTAAACTCAT-3′, Reverse—5′- TGTCTCTCTGTGACCATAGCC-3′; Human NR4A3: Forward—5′- CTTTGCAACGCTGACGGTG-3′, Reverse—5′-CGACTCATGGGAGAGCACAG-3′; Human SERPINE1: Forward—5′-GGAGAAACCCAGCAGCAGATT-3′, Reverse—5′-CTGTGGTGCTGATCTCATCCTT-3′; Human STAT1: Forward—5′-GCACGCTGCCAATGATGTTT-3′, Reverse—5′-ACATCTGGATTGGGTCTTCCTG-3′; Human ELK1: Forward—5′-CTTCACGGGATGGTGGTGAA-3′, Reverse—5′-CCGGCTGAGCTTGTCGTAAT-3′; Human JUNB: Forward—5′-CCCTACCGGAGTCTCAAAGC-3′, Reverse—5′-TGCTGTTGGGGACAATCAGG-3′; Human DUSP1: Forward—5′-TCCCAACCATTTTGAGGGTCA-3′, Reverse—5′-AAACACCCTTCCTCCAGCATT-3′; Human FOS: Forward—5′-TTACTACCACTCACCCGCAG-3′, Reverse—5′-GCAGTGACCGTGGGAATGAA-3′; Human GAPDH: Forward—5′-TATAAATTGAGCCCGCAGCC-3′, Reverse—5′-CCCAATACGACCAAATCCGTTG-3′; and Human β-actin: Forward—5′-CATGTTTGAGACCTTCAACACCCC-3′, Reverse—5′-GCCATCTCCTGCTCGAAGTCTAG-3′.

### 2.4. Cell Proliferation Assay

Cell proliferation assay was performed using Cell Counting Kit-8 (CCK-8; Dojindo Molecular Technologies Inc., Kumamoto, Japan) according to the manufacturer’s protocol. Briefly, 7.5 × 10^3^ cells per well were seeded on 96-well plates and cultured in media with or without 8 μM tamoxifen. The cells were stained with 10 μL of 2-(2-methoxy-4-nitrophenyl)-3-(4-nitrophenyl)-5-(2,4-disulfophenyl)-2H-tetrazolium, monosodium salt (WST-8), for 3 h at 37 °C in a 5% CO_2_ incubator on designated days. Absorbance was measured using an ELISA reader (Softmax Pro., Molecular Devices, San Jose, CA, USA) at 450 nm. Tamoxifen treatment was refreshed every 24 h to maintain a constant concentration.

### 2.5. Spheroid Formation Assay

Spheroid cultures were performed according to the previous procedure with minor changes [30]. Briefly, 1 × 10^4^ cells/mL were counted and resuspended carefully using a 25 G syringe needle to obtain a single-cell suspension. The cells were pelleted, washed with cold PBS, and syringe-filtered again to ensure a single-cell suspension. Cells were plated onto Ultra-Low attachment 6-well plates (Corning, NY, USA) with 2 mL DMEM/F12 media (WelGENE Inc., Daegu, Korea) supplemented with 1% PSA, 2% B27, 10 ng/mL FGFb, 20 ng/mL EGF, 5 μg/mL insulin, and 4 μg/mL heparin. Cells were maintained at 37 °C in a 5% CO_2_ incubator on designated days. The number of spheroids with a diameter greater than 50 μm was regularly counted.

### 2.6. Invasion and Migration Assays

Invasion assay was performed using Matrigel^TM^ (BD, Franklin Lakes, NJ, USA), as previously described [31], while migration assay was carried out using the same method in the absence of Matrigel^TM^. Approximately 5 × 10^4^ cells were seeded in each chamber and incubated at 37 °C for 48 h. Invasive and migrating cells were stained with fluorochrome 4′,6-diamidino-2-phenylindole (DAPI), and observed via fluorescence microscopy. The acquired images were analyzed using the ImageJ software.

### 2.7. Apoptosis Assay by Annexin V-FITC/Propidium Iodide (PI) Staining

Apoptotic cells were determined using the EzWay^TM^ Annexin V-FITC (Komabiotech, Seoul, Korea) apoptosis detection kit. Approximately 5 × 10^5^ cells were seeded and incubated for 24 h with 15 μM tamoxifen. Subsequently, the cells were harvested, washed with cold PBS, and stained with Annexin V-FITC and PI. Stained cells were analyzed using BD FACS LSRII (BD Biosciences, San Jose, CA, USA), and data were acquired using BD FACSDiva^TM^ software.

### 2.8. Nuclear and Cytoplasmic Protein Fractionation

For nuclear and cytoplasmic protein fractionation, cells were lysed in cold buffer A (10 mM HEPES, pH 7.9, 10 mM KCl, 0.1 mM EDTA, 1 mM DTT, protease inhibitor) for 5 min on ice. NP-40 was then added to give a final concentration of 0.6%. The cytoplasmic protein of the total cell lysate was obtained by centrifugation for 30 s at 4 °C. The nuclear cell pellet was washed with cold PBS and lysed in cold buffer C (20 mM HEPES, pH 7.9, 0.4 M NaCl, 1 mM EDTA, 1 mM DTT, protease inhibitor) for 15 min on ice. Nuclear proteins were harvested by centrifugation for 15 min at 4 °C, and protein extracts were analyzed using western blotting.

### 2.9. Immunocytochemisty

For immunocytochemistry, cells were fixed with 4% paraformaldehyde in PBS for 15 min and rinsed with cold PBS. Cell permeabilization was performed using 0.25% Triton X-100 in PBS for 10 min. The cells were then blocked with 5% bovine serum albumin for 1 h. After blocking, cells were incubated with primary antibody against NR4A1 (Abcam, ab13851, Cambridge, UK) at 4 °C overnight. The cells were subsequently washed with PBS and incubated with goat anti-rabbit IgG Alexa Fluor 594 (Abcam, ab150080, Cambridge, UK) for 1 h in the dark. DAPI was added to the cells, which were then incubated for 20 min. Fluorescence images were acquired using a Zeiss LSM 700 confocal microscope (Carl Zeiss, Jena, Germany).

### 2.10. Western Blot Analysis

All breast cancer cell lines were treated under the appropriate conditions and lysed in NP-40 lysis buffer, after which their protein concentrations were estimated using the Pierce BCA Protein Assay Kit (Thermo Scientific, Waltham, MA, USA). Each lysate was separated by 8–12% SDS polyacrylamide gel electrophoresis and then transferred to Immobilon-P PVDF transfer membranes (Merck Millipore, Burlington, MA, USA). Immunoreactive bands were detected using suitable primary antibodies and the appropriate HRP–conjugated secondary antibodies. Bands were visualized using the SuperSignal West Pico Chemiluminescent Substrate Kit (Thermo Scientific, Waltham, MA, USA). Anti-NR4A1 (ab13851, Abcam, Cambridge, UK), anti-Caspase-7 (#12827, Cell Signaling Technology, Danvers, MA, USA), anti-Caspase-9 (#9508, Cell Signaling), anti-PARP (#9542, Cell Signaling), anti-Src (#2110, Cell Signaling), anti-phospho-Src (#6943, Cell Signaling), anti-MEK1/2 (#4694, Cell Signaling), anti-phospho-MEK1/2 (#9154, Cell Signaling), anti-ERK1/2 (#9102, Cell Signaling), anti-phospho-ERK1/2 (#9101, Cell Signaling), anti-HDAC1 (ab7028, Abcam), and anti-β-actin (ab6276, Abcam) were used to detect proteins.

### 2.11. In Silico Analysis

#### 2.11.1. Gene Expression Omnibus (GEO) Dataset

The GEO dataset (GSE1378) obtained from the GEO database (http://www.ncbi.nlm.nih.gov/geo (accessed on 18 June 2021)) was used to assess NR4A1 mRNA levels in luminal A type breast cancer cohorts treated with tamoxifen monotherapy.

#### 2.11.2. Kaplan-Meier Survival Analysis

The Kaplan-Meier plotter (http://www.kmplot.com (accessed on 18 June 2021)) was used to assess the effect of genes on the survival rate of human breast cancer samples. To investigate the prognostic value of NR4A family genes, patient samples were classified into low- and high-expression groups using median as the cutoff value.

#### 2.11.3. cBioportal

The web-available database cBioportal (http://www.cbioportal.org (accessed on 18 June 2021)) was used to evaluate the molecular profile changes of NR4A1 in breast cancer tissues. Patient sample data were obtained from the Breast Invasive Carcinoma (TCGA, Nature 2012).

#### 2.11.4. Gene Ontology Functional Enrichment Analysis

Gene ontology functional enrichment analysis was performed for differentially expressed genes (DEGs) according to NR4A1 mRNA levels using the DAVID database (version DAVID 6.8; http://david.ncifcrf.gov (accessed on 18 June 2021)) to annotate biological processes. Both upregulated and downregulated DEGs were used, and statistical significance was set at *p* < 0.01.

#### 2.11.5. Gene Set Enrichment Analysis (GSEA)

GSEA was performed to evaluate potential gene sets and pathways enriched in breast cancer tissue samples with high and low expression of NR4A1 obtained from the cBioportal database. The GSEA software was installed from the Broad Institute online website (https://www.gsea-msigdb.org/gsea/msigdb/index.jsp (accessed on 18 June 2021)). The enriched gene sets with a *p*-value < 0.05 and FDR *q*-value < 0.05 were considered statistically significant.

### 2.12. Statistical Analysis

Data are expressed as the mean ± standard error of the mean of at least three different experiments. Statistical differences were determined using the paired Student’s *t*-test. Statistical significance was set at *p* < 0.05.

## 3. Results

### 3.1. NR4A1 Is Associated with Tamoxifen Sensitivity in ER-Positive Breast Cancer

**Figure 1:**
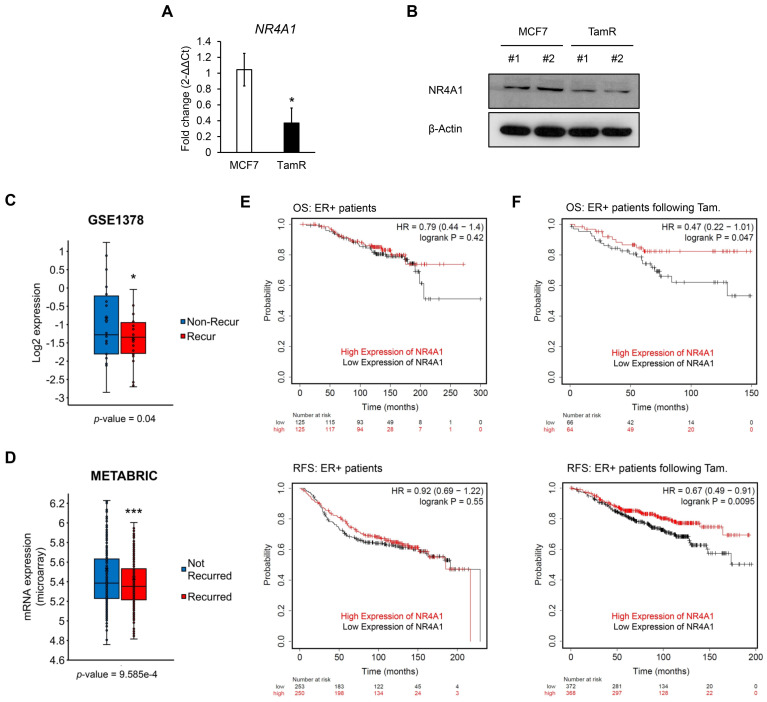
NR4A1 is associated with tamoxifen sensitivity in ER-positive breast cancer. mRNA (**A**) and protein (**B**) expression levels of NR4A1 in MCF7 and TamR breast cancer cells from two different passages. (**C**) The mRNA expression levels of NR4A1 in patients with luminal A type breast cancer and recurred patients treated with tamoxifen monotherapy for 5 years (GSE1378) (n = 52, *p* = 0.04) are shown using log2 expression values. (**D**) The mRNA expression levels of NR4A1 in patients with ER-positive breast cancer and recurred patients treated with hormone therapy (METABRIC) (n = 1079, *p* = 9.585 × 10^−4^) are shown using log2 expression values. Data was retrieved from cBioportal. (**E**) Kaplan-Meier analysis curves of overall survival (OS; upper panel) (n = 250, *p* = 0.42) and recurrence-free survival (RFS; lower panel) (n = 503, *p* = 0.55) rates of patients with ER-positive breast cancer receiving no treatment. (**F**) Kaplan-Meier analysis curves of OS (upper panel; n = 130, *p* = 0.047) and RFS (lower panel; n = 740, *p* = 0.0095) rates of patients with ER-positive breast cancer following tamoxifen therapy. The NR4A1 (211143_x_at) probe was used and analyzed to detect survival rates. * *p* < 0.05 and *** *p* < 0.001.

To investigate NR4A1 gene expression in tamoxifen-resistant (TamR) breast cancer cells, we examined the mRNA and protein expression levels of NR4A1 in MCF7 and TamR cells using qRT-PCR and western blotting. Our results showed that the mRNA and protein levels of NR4A1 were significantly downregulated in TamR cells compared to those in MCF7 and T47D cells (Figure 1A,B and Figure S1A,B). We also compared the expression of NR4A1 in clinical breast cancer patient samples retrieved from a publicly available dataset (GSE1378, METABRIC). The expression levels of NR4A1 decreased in patients with recurred (tamoxifen-resistant) luminal A type breast cancer treated with tamoxifen therapy (GSE1378) and hormone therapy (METABRIC) compared to those in patients with non-recurred (tamoxifen-sensitive) luminal A type breast cancer (Figure 1C,D).

Further, we examined whether the elevated NR4A1 expression was associated with an increased survival rate in human breast cancer using NR4A1 gene expression. To assess the clinical relevance between NR4A1 expression and survival rate, we analyzed the survival of patients with ER-positive breast cancer using Kaplan-Meier survival analysis. High expression of NR4A1 correlated with good overall survival (OS) and relapse-free survival (RFS) in patients with ER-positive breast cancer following tamoxifen therapy, although there was no significant association in untreated patients (Figure 1E,F). The NR4A family genes NR4A2 and NR4A3 showed no significant difference between the mRNA levels of MCF7 and TamR cells, although high NR4A3 level represented favorable OS and RFS in patients treated with tamoxifen therapy, but not NR4A2 level (Figure S2A–F). Taken together, these results show that NR4A1 is associated with tamoxifen sensitivity in human ER-positive breast cancer.

### 3.2. NR4A1 Suppresses Proliferation, Migration, and Invasion Ability, While Increasing Ttamoxifen-Induced Apoptosis in TamR Cells

**Figure 2:**
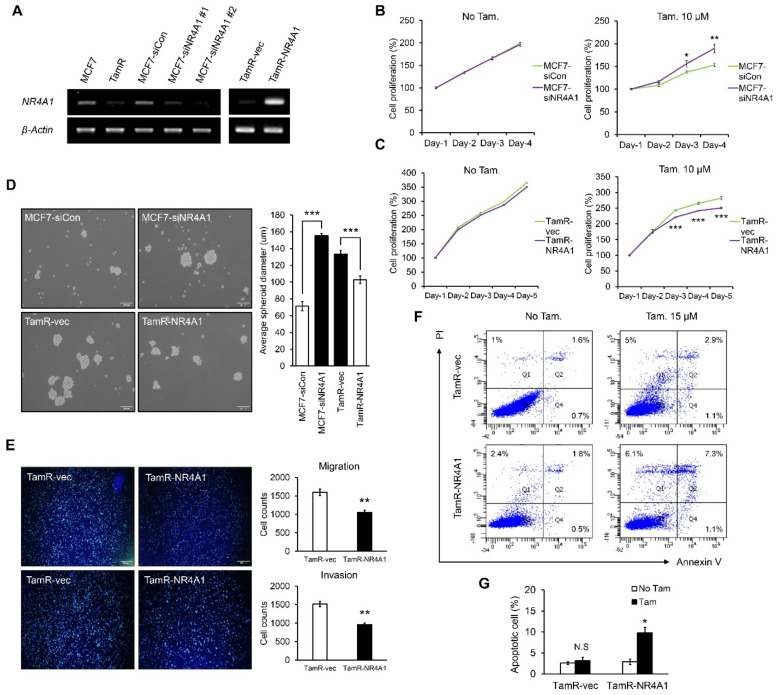
NR4A1 suppresses proliferation, migration, and invasion, while increasing tamoxifen-induced apoptosis in TamR cells. (**A**) mRNA expression levels of NR4A1 in MCF7, TamR, MCF7 cells transfected with siControl (siCon) or NR4A1 siRNA (siNR4A1), and TamR cells transfected with pcDNA3 (empty vector) or pcDNA3-NR4A1. (**B**) Cell proliferation curves of siCon/siNR4A1 MCF7 cells untreated (No Tam.) or treated with 10 μM tamoxifen (Tam.) for 4 days. (**C**) Cell proliferation curves of TamR-vec and TamR-NR4A1 cells untreated (No Tam.) or treated with 10 μM tamoxifen (Tam.) for 5 days. (**D**) Representative images of breast cancer spheroids growth of MCF7 cells transfected with siCon or siNR4A1, and TamR cells transfected with empty vector or pcDNA3-NR4A1 (left panel). Spheroid formation was analyzed after 6 days. Quantification of average spheroid diameter (right panel) is represented on a bar graph. (**E**) Matrigel invasion and migration assays in empty vector transfected TamR and stable NR4A1-overexpressing TamR cells. Migrating and invasive cells stained with DAPI were calculated using imageJ. (**F**) FACS analysis of apoptotic cell death in TamR-vec and TamR-NR4A1 cells untreated or treated with 15 μM tamoxifen. Apoptotic cells were stained by Annexin V/PI. (**G**) Percentage of apoptotic cells from (**F**) are represented on a bar graph. * *p* < 0.05, ** *p* < 0.01, and *** *p* < 0.001.

To confirm tamoxifen resistance in our cell line, we compared cell proliferation between MCF7 and TamR cells using the CCK-8 assay. TamR cells were resistant to tamoxifen treatment (Figure S3A). To investigate the functional role of NR4A1 in TamR cells, we confirmed the ectopic overexpression of NR4A1 in TamR cells and knockdown of NR4A1 in MCF7 cells via RT-PCR and western blot (Figure 2A and Figure S4). There was no difference in cell proliferation when NR4A1 was knocked down in MCF7 cells in the absence of tamoxifen (No Tam.). Interestingly, NR4A1 knockdown increased tamoxifen resistance in MCF7 cells treated with tamoxifen for 4 days (Figure 2B). Consistent with the results of NR4A1 knockdown, overexpression of NR4A1 significantly restored sensitivity to tamoxifen in TamR cells (Figure 2C). These results suggest that NR4A1 expression regulates tamoxifen sensitivity in ER-positive breast cancer cells.

We compared tumor formation ability using 3-D spheroid formation assay to mimic the characteristics of human tumors. We confirmed that TamR cells represent higher spheroid formation ability compared to MCF7 cells (Figure S3B). To confirm that this spheroid formation was due to the expression of NR4A1 in ER-positive breast cancer cells, NR4A1-depleted MCF7 cells and NR4A1-overexpressing TamR cells were used to assess the spheroid formation abilities. A significant increase of spheroid in NR4A1 knockdown MCF7 cells compared to control cells was examined, and overexpression of NR4A1 significantly inhibited the size of spheroid in TamR cells (Figure 2D). These results showed that NR4A1 expression significantly suppressed solid tumor-like properties in ER-positive breast cancer.

Because tamoxifen-resistant breast cancer cells have migratory and invasive properties [32], we confirmed that TamR cells exhibit higher migratory and invasive abilities than those of MCF7 cells through transwell migration and invasion assays (Figure S3C). We then evaluated the effect of NR4A1 expression on the migration and invasion abilities of TamR cells. As shown in Figure 2E, NR4A1 overexpression significantly reduced these abilities in TamR cells.

Since it is known that NR4A1 induces cell apoptosis in several tumor types [33], we investigated whether the expression of NR4A1 re-sensitizes TamR cells to tamoxifen treatment by increasing apoptotic ability. Flow cytometry analysis was performed using annexin V/PI staining to compare the apoptotic levels between MCF7 and TamR cells in the absence and presence of tamoxifen. High-dose tamoxifen (15 μM) treatment induced apoptosis in MCF7 cells, while there were insignificant changes in TamR cells (Figure S3D,E). In addition, overexpression of NR4A1 in TamR cells (TamR-NR4A1) significantly increased the percentage of apoptotic cell death compared to that in control cells (TamR-vec) when tamoxifen was added (Figure 2F,G). These results demonstrated that NR4A1 overexpression enhanced tamoxifen-induced apoptotic ability of tamoxifen-resistant ER-positive breast cancer cells. Thus, NR4A1 inhibits cell proliferation, migration, and invasion ability, while enhancing tamoxifen-induced apoptosis in TamR cells.

### 3.3. NR4A1 Localized in the Cytoplasm Affects Apoptosis

**Figure 3:**
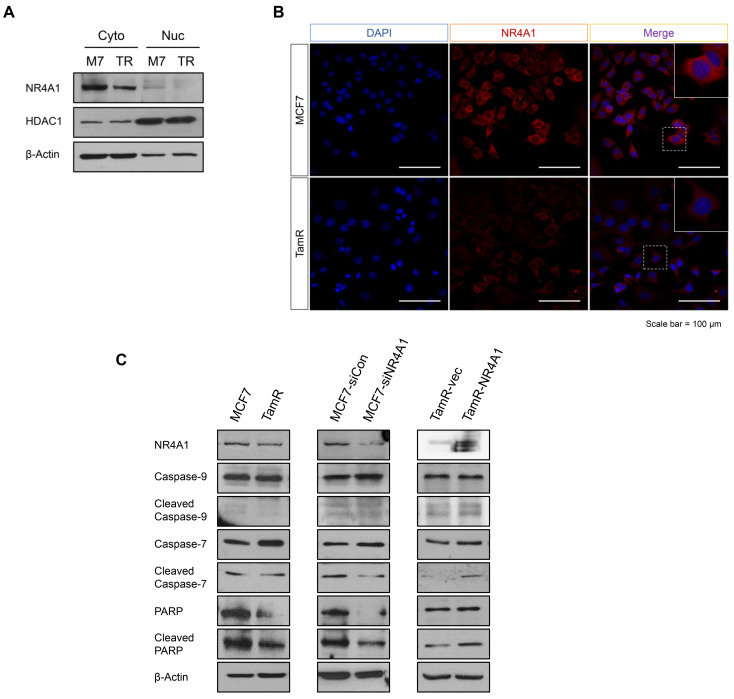
NR4A1 localized in the cytoplasm influences apoptosis. (**A**) Western blot analysis of cytoplasmic and nuclear NR4A1 proteins in MCF7 and TamR cells. (**B**) Representative images of immunofluorescence for NR4A1 localization in MCF7 and TamR cells. Scale bar = 100 μm. (**C**) Western blot analysis for apoptosis-related proteins in MCF7, TamR, MCF7 cells transfected with scramble (negative) control (siCon), NR4A1 siRNA (siNR4A1), and TamR cells transfected with pcDNA3 and pcDNA3-NR4A1.

Since NR4A1 has different functions depending on subcellular localization in various types of cancer model [34], we compared the distribution and subcellular localization of NR4A1 between MCF7 and TamR cells by nuclear and cytoplasmic fractionation. Our results showed that NR4A1 is primarily localized in the cytoplasm in both cells and up-regulated in MCF7 than TamR cells (Figure 3A). Immunocytochemistry analysis also revealed that NR4A1 predominantly localizes in the cytoplasm of MCF7 cells (Figure 3B). Although NR4A1 also existed slightly in the nuclear fraction, we focused on the cytoplasm, as NR4A1 was more abundant in the cytoplasm. It has been reported that NR4A1, which translocates from the nucleus to the cytoplasm, releases cytochrome c and induces apoptosis [35]. Thus, we hypothesized that high expression of cytoplasmic NR4A1 in MCF7 cells might cause apoptosis and explored the expression of pro-apoptotic genes by western blotting. Results showed that the protein levels of cleaved caspase-7, -9, and PARP, and uncleaved PARP were higher in MCF7 cells than in TamR cells. When NR4A1 was knocked down in MCF7 cells, the levels of cleaved caspase-7, cleaved PARP, and uncleaved PARP decreased, while the level of cleaved caspase-9 did not change. Conversely, when NR4A1 was overexpressed in TamR, the cleaved forms of caspase-7, -9, and PARP increased, although the uncleaved forms did not change (Figure 3C). Additionally, we performed immunocytochemistry to confirm the subcellular localization of NR4A1 when NR4A1 was overexpressed in TamR and found that NR4A1 was mainly distributed in the cytoplasm (Figure S5). These results confirmed that NR4A1 localized in the cytoplasm plays an essential role in inducing the expression of apoptosis-related factors.

### 3.4. NR4A1 Alters Tamoxifen Sensitivity in ER-Positive Breast Cancer Cells by Suppressing ERK Signaling Pathway

**Figure 4:**
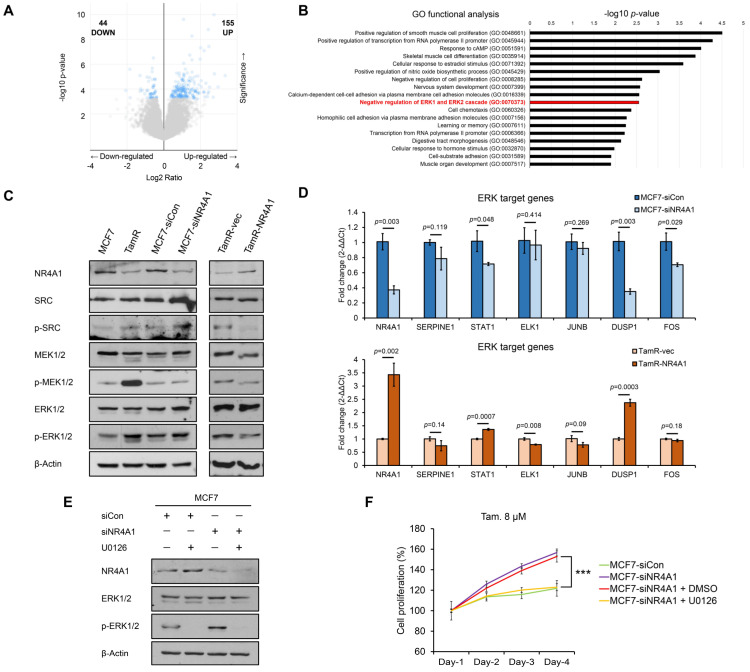
NR4A1 alters tamoxifen sensitivity in ER-positive breast cancer cells by suppressing the ERK signaling pathway. (**A**) The volcano plot of significantly up- and down-regulated genes according to mRNA expression profile of NR4A1 gene (Student’s *t*-test *p* < 0.05 and Benjamini-Hochberg procedure *q* < 0.05). (**B**) Gene ontology functional enrichment analysis showing biological processes enriched in 199 genes regulated by NR4A1. (**C**) Western blot analysis of ERK signaling pathway-related protein in MCF7, TamR, MCF7 cells transfected with scramble (negative) control (siCon), NR4A1 siRNA (siNR4A1), and TamR cells transfected with pcDNA3 and pcDNA3-NR4A1. (**D**) mRNA expression levels of ERK target genes in MCF7 cells transfected with scramble (negative) control (siCon) and NR4A1 siRNA (siNR4A1), and TamR cells transfected with pcDNA3 and pcDNA3-NR4A1. (**E**) Western blot analysis of NR4A1, ERK1/2, and p-ERK1/2 proteins in MCF7-siCon and siNR4A1 cells following treatment with U0126 or DMSO (control) (**F**) Cell proliferation curves of MCF7-siCon and siNR4A1 cells treated with U0126 or DMSO (control) under the presence of 8 μM tamoxifen (Tam.) for 4 days. *** *p* < 0.001.

To investigate the molecular mechanism by which NR4A1 regulates tamoxifen sensitivity, we performed gene expression profiling using the breast cancer patient dataset retrieved from cBioportal. We ultimately identified 199 DEGs according to NR4A1 mRNA levels (Figure 4A). To determine the biological function of the target genes, DEGs were subjected to gene ontology functional enrichment analysis using the DAVID database. Results showed that these NR4A1 target genes were significantly enriched in the negative regulation of ERK1 and ERK2 cascade (Figure 4B). Moreover, GSEA conducted between the NR4A1 high and low groups revealed that high mRNA levels of NR4A1 are correlated with the negative regulation of MAPK cascade (Figure S6A,B). Furthermore, previous studies have reported that cytoplasmic NR4A1 is associated with ERK signaling and a good prognosis [36]. This evidence raises the possibility that NR4A1 genes may contribute to the negative regulation of the ERK signaling pathway in breast cancer. Therefore, we examined the expression levels of proteins related to the ERK signaling pathway. First, the results demonstrated that the protein levels of p-SRC, p-MEK1/2, and p-ERK1/2 were higher in TamR cells than in MCF7 cells. In addition, western blot analysis confirmed that NR4A1 could downregulate the levels of p-SRC, p-MEK1/2, and p-ERK1/2 (Figure 4C). Because NR4A1 changes the level of phosphorylated ERK protein, which is functional, we assumed that NR4A1 would also change the expression of target genes that are directly regulated by ERK. Therefore, the transcription levels of several ERK target genes were confirmed by quantitative real-time PCR. Results showed that ERK target genes such as STAT1 and DUSP1 were also regulated according to the expression of NR4A1 (Figure 4D). The ERK signaling pathway is known to play an important role in the tamoxifen-resistant phenotype [26]. To evaluate whether the induction of ERK signaling is necessary for silenced-NR4A1-mediated tamoxifen resistance, we treated MCF7-siNR4A1 cells with U0126, an ERK1/2 inhibitor, and performed CCK-8 assay. Results showed that U0126 could reverse the ability of cell proliferation, which was enhanced by NR4A1 knockdown (Figure 4E). Additionally, we measured the expression level of p-ERK1/2 after treatment with U0126. As shown in Figure 4F, the inhibitor treatment efficiently inhibited the phosphorylation of ERK1/2, which was increased following NR4A1 knockdown without affecting the total ERK1/2 level. Taken together, our data suggest that NR4A1 regulates tamoxifen sensitivity by inhibiting the ERK signaling pathway in ER-positive breast cancer.

## 4. Discussion

Here, we demonstrated that NR4A1, whose expression was downregulated in TamR cells and patients with recurrent breast cancer treated with tamoxifen, may be a new prognostic factor and therapeutic target for tamoxifen resistance. In our Kaplan-Meier analysis, we found that high expression of NR4A1 in patients with ER-positive breast cancer treated with tamoxifen associates with a good prognosis. Through gain- and loss-of-function study, the subsequent upregulation of NR4A1 expression enhanced tamoxifen sensitivity, thereby regulating cell proliferation, migration, invasion, and apoptosis in tamoxifen-resistant cells. More importantly, high cytoplasmic NR4A1 contributes to apoptosis by inducing the expression of pro-apoptotic factors in breast cancer cells. Mechanistically, we revealed that NR4A1 restored tamoxifen sensitivity by inhibiting the ERK signaling pathway in tamoxifen-resistant breast cancer cells.

NR4A1 is an early response gene promptly induced by various cellular stimuli and plays an important role in various physiological processes and diseases, including cancer [9]. Several studies have shown that NR4A1 promotes or inhibits tumor progression depending on the tumor type, cell-specific context, and various external stimuli. NR4A1 expression has been previously reported to be upregulated in non-small-cell lung carcinoma tissues compared to that in normal tissues, and high NR4A1 level has shown an oncogenic role as a prognostic marker for predicting adverse clinical outcomes in non-small-cell lung carcinoma [37]. Additionally, NR4A1 expression suppresses cancer growth and progression in triple-negative breast cancer (TNBC) in vitro and in vivo, showing its role as a tumor suppressor [20]. Moreover, recent studies have shown that NR4A1 induces TNFα-mediated apoptosis sensitivity in human gastric cancer [38] and improves cisplatin resistance in ovarian cancer [39], supporting that NR4A1 plays a crucial role in drug therapy sensitivity in a variety of tumor models. Nevertheless, the exact role of NR4A1 in tamoxifen resistance in breast cancer has not been elucidated. In the present study, high levels of NR4A1 were found to be associated with a favorable prognosis and tamoxifen sensitivity in patients with ER-positive breast cancer treated with tamoxifen. Moreover, tamoxifen-resistant cell line (TamR) used in this study showed downregulated NR4A1 expression, whereas other NR4A factors, NR4A2 and NR4A3, did not differ in expression between MCF7 and TamR cells. Overexpression of NR4A1 restored the sensitivity to tamoxifen and attenuated proliferation, migration, and invasion; however, it enhanced tamoxifen-induced apoptosis in TamR cells. This phenomenon has also been observed in previous studies that NR4A1 reduces migration in both normal and breast cancer cells [40] and mediates apoptosis in aggressive B-cell lymphoma [33], indicating that NR4A1, which regulates cellular processes in response to tamoxifen, could suppress the acquisition of a tamoxifen-resistant phenotype. Thus, NR4A1 could prove useful as a predictor of tamoxifen responsiveness and therapeutic target to overcome tamoxifen resistance in ER-positive breast cancer.

Localization of NR4A1 protein in the cell is crucial for understanding its biological effects, and subcellular mislocalization of proteins can work as a molecular therapeutic intervention [41]. The function of NR4A1 is influenced not only by expression but also by its localization within the cell. NR4A1 present in the nucleus is translocated to the cytoplasm in response to several apoptosis-inducing agents, where it binds to Bcl-2 and forms a pro-apoptotic complex, inducing cytochrome c release and cell apoptosis through a p53-independent intrinsic pathway [35]. In this study, surprisingly, we found that NR4A1 is mainly located in the cytoplasm of both MCF7 and TamR breast cancer cells and promoted the expression of apoptosis-related molecules, including caspase-7, caspase-9, and PARP. Similarly, several studies have also shown that NR4A1 in the cytoplasm induces apoptosis mediated by apoptotic-associated molecules [38,42]. Hence, its reduced expression in tamoxifen-resistant tumor cells may contribute to apoptosis and cell survival, and translocation of NR4A1 to the cytoplasm from the nucleus by various agents, including cytosporone B and C-DIM [43,44] may serve as a unique therapeutic strategy. However, it is still unclear whether nuclear localization of NR4A1 through drugs that block nuclear export of NR4A1 confers tamoxifen resistance or tumor progression, thus requiring further investigation.

According to previous reports, cytoplasmic NR4A1 is associated with the ERK signaling pathway [36]. ERK signaling is a pathway that plays a crucial role in tamoxifen resistance as well as cellular signaling in various cancers, including breast cancer [26]. In addition, activated ERK signaling reduces responsiveness and effectiveness of tamoxifen treatment in patients with ER-positive breast cancer and affects poor prognosis [27,28]. These studies suggest that ERK acts as a prognostic marker and can serve as an indicator of the effectiveness of tamoxifen treatment in breast cancer patients.

In the present study, through in silico gene functional analysis and in vitro experiments, we showed that NR4A1 not only reduces the expression of factors related to ERK signaling but also regulates the mRNA expression of ERK downstream target genes (STAT1 and DUSP1), implying that ERK signaling may represent a general target pathway of NR4A1. We further confirmed that the enhanced proliferation ability induced by NR4A1 knockdown is greatly impaired after treatment with an ERK inhibitor. Similarly, a previous study reported that NR4A1 mitigates the level of active ERK1/2 and ERK inhibition following U0126 treatment in NR4A1-overexpressing normal breast epithelial cells suppresses cell migration [40]. These results support our hypothesis that deactivation of the ERK signaling pathway is responsible for NR4A1-induced tamoxifen sensitivity. Accordingly, the regulation of ERK signaling by NR4A1 revealed in this study is a potential therapeutic target as well as a biomarker for treating tamoxifen resistance.

Our study is the first to reveal that NR4A1 is a marker associated with tamoxifen sensitivity. In this study, NR4A1 induced sensitivity to tamoxifen by regulating cell proliferation, migration, invasion, and apoptosis in ER-positive breast cancer cells. In addition, translocation of NR4A1 to the cytoplasm from the nucleus localized in the cytoplasm activated apoptotic molecules. Further, NR4A1 inhibited the ERK signaling cascade, which in turn regulated ERK target genes, resulting in attenuated tamoxifen resistance in TamR cells. Collectively, we have shown that NR4A1 is a crucial mediator of apoptosis depending on their cellular location. our study suggests the NR4A1/ERK signaling axis plays an important role in overcoming tamoxifen resistance and could be a novel therapeutic target for attenuating tamoxifen resistance in patients with ER-positive breast cancer.

## Data Availability

The raw data presented in this study can be obtained from the corresponding author.

## Supplementary Materials

The following are available online at https://www.mdpi.com/article/10.3390/cells10071633/s1, Figure S1: NR4A1 is down-regulated in T47D TamR cells; Figure S2: NR4A2 and NR4A3 are not associated with tamoxifen sensitivity in ER-positive breast cancer; Figure S3: TamR cells are more resistant to tamoxifen compared to MCF7 cells; Figure S4: The efficiency of NR4A1 knockdown in MCF7 cells; Figure S5: NR4A1 localizes in the cytoplasm following NR4A1 overexpression in TamR cells; Figure S6: High NR4A1 level is related to negative regulation of ERK1, ERK2, and MAPK signaling pathways.

## References

[1] DeSantis C.E., Ma J., Gaudet M.M., Newman L.A., Mph K.D.M., Sauer A.G., Jemal A., Siegel R.L. (2019). Breast cancer statistics, 2019. CA A Cancer J. Clin..

[2] Fragomeni S.M., Sciallis A., Jeruss J.S. (2018). Molecular Subtypes and Local-Regional Control of Breast Cancer. Surg. Oncol. Clin. N. Am..

[3] Ali S., Coombes R.C. (2002). Endocrine-responsive breast cancer and strategies for combating resistance. Nat. Rev. Cancer.

[4] Lumachi F., Luisetto G., Basso S.M., Basso U., Brunello A., Camozzi V. (2011). Endocrine Therapy of Breast Cancer. Curr. Med. Chem..

[5] Early Breast Cancer Trialists’ Collaborative Group (EBCTCG) (2005). Effects of chemotherapy and hormonal therapy for early breast cancer on recurrence and 15-year survival: An overview of the randomised trials. Lancet.

[6] Normanno N., Di Maio M., De Maio E., De Luca A., De Matteis A., Giordano A., Perrone F. (2005). Mechanisms of endocrine resistance and novel therapeutic strategies in breast cancer. Endocrine-Related Cancer.

[7] Herring J.A., Elison W.S., Tessem J.S. (2019). Function of Nr4a Orphan Nuclear Receptors in Proliferation, Apoptosis and Fuel Utilization Across Tissues. Cells.

[8] Mohan H.M., Aherne C.M., Rogers A., Baird A.W., Winter D.C., Murphy E.P. (2012). Molecular Pathways: The Role of NR4A Orphan Nuclear Receptors in Cancer. Clin. Cancer Res..

[9] Zhang L., Wang Q., Liu W., Liu F., Ji A., Li Y. (2018). The Orphan Nuclear Receptor 4A1: A Potential New Therapeutic Target for Metabolic Diseases. J. Diabetes Res..

[10] McMorrow J., Murphy E.P. (2011). Inflammation: A role for NR4A orphan nuclear receptors?. Biochem. Soc. Trans..

[11] Cho S.D., Yoon K., Chintharlapalli S., Abdelrahim M., Lei P., Hamilton S., Khan S., Ramaiah S.K., Safe S. (2007). Nur77 Agonists Induce Proapoptotic Genes and Responses in Colon Cancer Cells through Nuclear Receptor–Dependent and Nuclear Receptor–Independent Pathways. Cancer Res..

[12] Zhao B.-X., Chen H.-Z., Du X.-D., Luo J., He J.-P., Wang R.-H., Wang Y., Wu R., Hou R.-R., Hong M. (2011). Orphan Receptor TR3 Enhances p53 Transactivation and Represses DNA Double-Strand Break Repair in Hepatoma Cells under Ionizing Radiation. Mol. Endocrinol..

[13] Hedrick E., Mohankumar K., Safe S. (2018). TGFβ-Induced Lung Cancer Cell Migration Is NR4A1-Dependent. Mol. Cancer Res..

[14] Li X.-X., Wang Z.-J., Zheng Y., Guan Y.-F., Yang P.-B., Chen X., Peng C., He J.-P., Ai Y.-L., Wu S.-F. (2018). Nuclear Receptor Nur77 Facilitates Melanoma Cell Survival under Metabolic Stress by Protecting Fatty Acid Oxidation. Mol. Cell.

[15] Ramirez-Herrick A.M., Mullican S.E., Sheehan A.M., Conneely O.M. (2011). Reduced NR4A gene dosage leads to mixed myelodysplastic/myeloproliferative neoplasms in mice. Blood.

[16] Mullican S.E., Zhang S., Konopleva M., Ruvolo V., Andreeff M., Milbrandt J., Conneely O.M. (2007). Abrogation of nuclear receptors Nr4a3 andNr4a1 leads to development of acute myeloid leukemia. Nat. Med..

[17] Crawford P.A., Sadovsky Y., Woodson K., Lee S.L., Milbrandt J. (1995). Adrenocortical function and regulation of the steroid 21-hydroxylase gene in NGFI-B-deficient mice. Mol. Cell. Biol..

[18] Zhou F., Drabsch Y., Dekker T.J.A., De Vinuesa A.G., Li Y., Hawinkels L.J.A.C., Sheppard K.-A., Goumans M.-J., Luwor R.B., De Vries C.J. (2014). Nuclear receptor NR4A1 promotes breast cancer invasion and metastasis by activating TGF-β signalling. Nat. Commun..

[19] Ye X.-F., Wu Q., Liu S., Lin X.-F., Zhang B., Wu J.-F., Cai J.-H., Zhang M.-Q., Su W.-J. (2004). Distinct role and functional mode of TR3 and RARα in mediating ATRA-induced signalling pathway in breast and gastric cancer cells. Int. J. Biochem. Cell Biol..

[20] Wu H., Bi J., Peng Y., Huo L., Yu X., Yang Z., Zhou Y., Qin L., Xu Y., Liao L. (2017). Nuclear receptor NR4A1 is a tumor suppressor down-regulated in triple-negative breast cancer. Oncotarget.

[21] Zhang W., Liu H.T. (2002). MAPK signal pathways in the regulation of cell proliferation in mammalian cells. Cell Res..

[22] Yuan J., Dong X., Yap J., Hu J. (2020). The MAPK and AMPK signalings: Interplay and implication in targeted cancer therapy. J. Hematol. Oncol..

[23] Santen R.J., Song R.X., McPherson R., Kumar R., Adam L., Jeng M.-H., Yue W. (2002). The role of mitogen-activated protein (MAP) kinase in breast cancer. J. Steroid Biochem. Mol. Biol..

[24] Salaroglio I.C., Mungo E., Gazzano E., Kopecka J., Riganti C. (2019). ERK is a Pivotal Player of Chemo-Immune-Resistance in Cancer. Int. J. Mol. Sci..

[25] Adeyinka A., Nui Y., Cherlet T., Snell L., Watson P., Murphy L.C. (2002). Activated mitogen-activated protein kinase expression during human breast tumorigenesis and breast cancer progression. Clin. Cancer Res..

[26] Xie F., Li Z., Wang N., Fang J., Huang J., Tian F., Li C. (2012). Role of PKC-ERK signaling in tamoxifen-induced apoptosis and tamoxifen resistance in human breast cancer cells. Oncol. Rep..

[27] Mueller H., Flury N., Eppenberger-Castori S., Kueng W., Eppenberger U. (2000). Potential prognostic value of mitogen-activated protein kinase activity for disease-free survival of primary breast cancer patients. Int. J. Cancer.

[28] Gee J.M., Robertson J.F., Ellis I.O., Nicholson R.I. (2001). Phosphorylation of ERK1/2 mitogen-activated protein kinase is associated with poor response to anti-hormonal therapy and decreased patient survival in clinical breast cancer. Int. J. Cancer.

[29] Yang S., Lee J.-Y., Hur H., Oh J.H., Kim M.H. (2018). Up-regulation of HOXB cluster genes are epigenetically regulated in tamoxifen-resistant MCF7 breast cancer cells. BMB Rep..

[30] Iglesias J.M., Beloqui I., Garcia-Garcia F., Leis O., Vázquez-Martín A., Eguiara A., Cufí S., Pavon A., Menendez J.A., Dopazo J. (2013). Mammosphere Formation in Breast Carcinoma Cell Lines Depends upon Expression of E-cadherin. PLoS ONE.

[31] Lee J.-Y., Hur H., Yun H.J., Kim Y., Yang S., Kim S.I., Kim M.H. (2015). HOXB5 Promotes the Proliferation and Invasion of Breast Cancer Cells. Int. J. Biol. Sci..

[32] Gooding A.J., Schiemann W.P. (2020). Epithelial–Mesenchymal Transition Programs and Cancer Stem Cell Phenotypes: Mediators of Breast Cancer Therapy Resistance. Mol. Cancer Res..

[33] Deutsch A.J.A., Rinner B., Wenzl K., Pichler M., Troppan K., Steinbauer E., Schwarzenbacher D., Reitter S., Feichtinger J., Tierling S. (2014). NR4A1-mediated apoptosis suppresses lymphomagenesis and is associated with a favorable cancer-specific survival in patients with aggressive B-cell lymphomas. Blood.

[34] Kolluri S.K., Bruey-Sedano N., Cao X., Lin B., Lin F., Han Y.-H., Dawson M.I., Zhang X.-K. (2003). Mitogenic Effect of Orphan Receptor TR3 and Its Regulation by MEKK1 in Lung Cancer Cells. Mol. Cell. Biol..

[35] Lin B., Kolluri S.K., Lin F., Liu W., Han Y.-H., Cao X., Dawson M.I., Reed J.C., Zhang X.-K. (2004). Conversion of Bcl-2 from Protector to Killer by Interaction with Nuclear Orphan Receptor Nur77/TR3. Cell.

[36] Fechter K., Feichtinger J., Prochazka K., Unterluggauer J.J., Pansy K., Steinbauer E., Pichler M., Haybaeck J., Prokesch A., Greinix H.T. (2018). Cytoplasmic location of NR4A1 in aggressive lymphomas is associated with a favourable cancer specific survival. Sci. Rep..

[37] Zhu B., Yang J.-R., Jia Y., Zhang P., Shen L., Li X.-L., Li J., Wang B. (2017). Overexpression of NR4A1 is associated with tumor recurrence and poor survival in non-small-cell lung carcinoma. Oncotarget.

[38] Yan H., Xiao F., Zou J., Qiu C., Sun W., Gu M., Zhang L. (2017). NR4A1-induced increase in the sensitivity of a human gastric cancer line to TNFα-mediated apoptosis is associated with the inhibition of JNK/Parkin-dependent mitophagy. Int. J. Oncol..

[39] Wilson A.J., Liu A.Y., Roland J., Adebayo O.B., Fletcher S.A., Slaughter J.C., Saskowski J., Crispens M.A., Jones H.W., James S. (2013). TR3 Modulates Platinum Resistance in Ovarian Cancer. Cancer Res..

[40] Alexopoulou A.N., Leao M., Caballero O.L., Da Silva L., Reid L., Lakhani S.R., Simpson A.J., Marshall J.F., Neville A.M., Jat P.S. (2010). Dissecting the transcriptional networks underlying breast cancer: NR4A1 reduces the migration of normal and breast cancer cell lines. Breast Cancer Res..

[41] Wang X., Li S. (2014). Protein mislocalization: Mechanisms, functions and clinical applications in cancer. Biochim. Biophys. Acta Bioenergy.

[42] He L., Yuan L., Yu W., Sun Y., Jiang D., Wang X., Feng X., Wang Z., Xu J., Yang R. (2020). A Regulation Loop between YAP and NR4A1 Balances Cell Proliferation and Apoptosis. Cell Rep..

[43] Zhan Y., Du X., Chen H., Liu J., Zhao B., Huang D., Li G., Xu Q., Zhang M., Weimer B.C. (2008). Cytosporone B is an agonist for nuclear orphan receptor Nur77. Nat. Chem. Biol..

[44] Lei P., Abdelrahim M., Cho S.D., Liu X., Liu X., Safe S. (2008). Structure-dependent activation of endoplasmic reticulum stress-mediated apoptosis in pancreatic cancer by 1,1-bis(3′-indoly)-1-(p-substituted phenyl)methanes. Mol. Cancer Ther..

