# Supplementary material for: NR4A1 Regulates Tamoxifen Resistance by Suppressing ERK Signaling in ER-Positive Breast Cancer
Source: Cells. 2021 Jun 29;10(7):1633. doi: 10.3390/cells10071633 (PMC8307977; doi:10.3390/cells10071633)
Supplement: Supplementary file 1 [file cells-10-01633-s001.zip › cells-1266902-Supplementary figures.pdf]

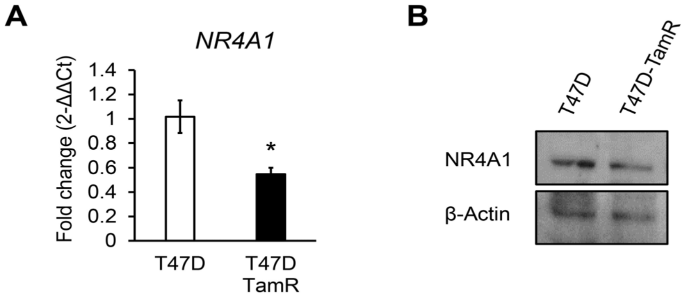

Figure S1. NR4A1 is downregulated in T47D TamR cells. RT-qPCR (A) and Western blot (B) analysis of NR4A1 in T47D and T47D-TamR cells. * $p < 0.05$.

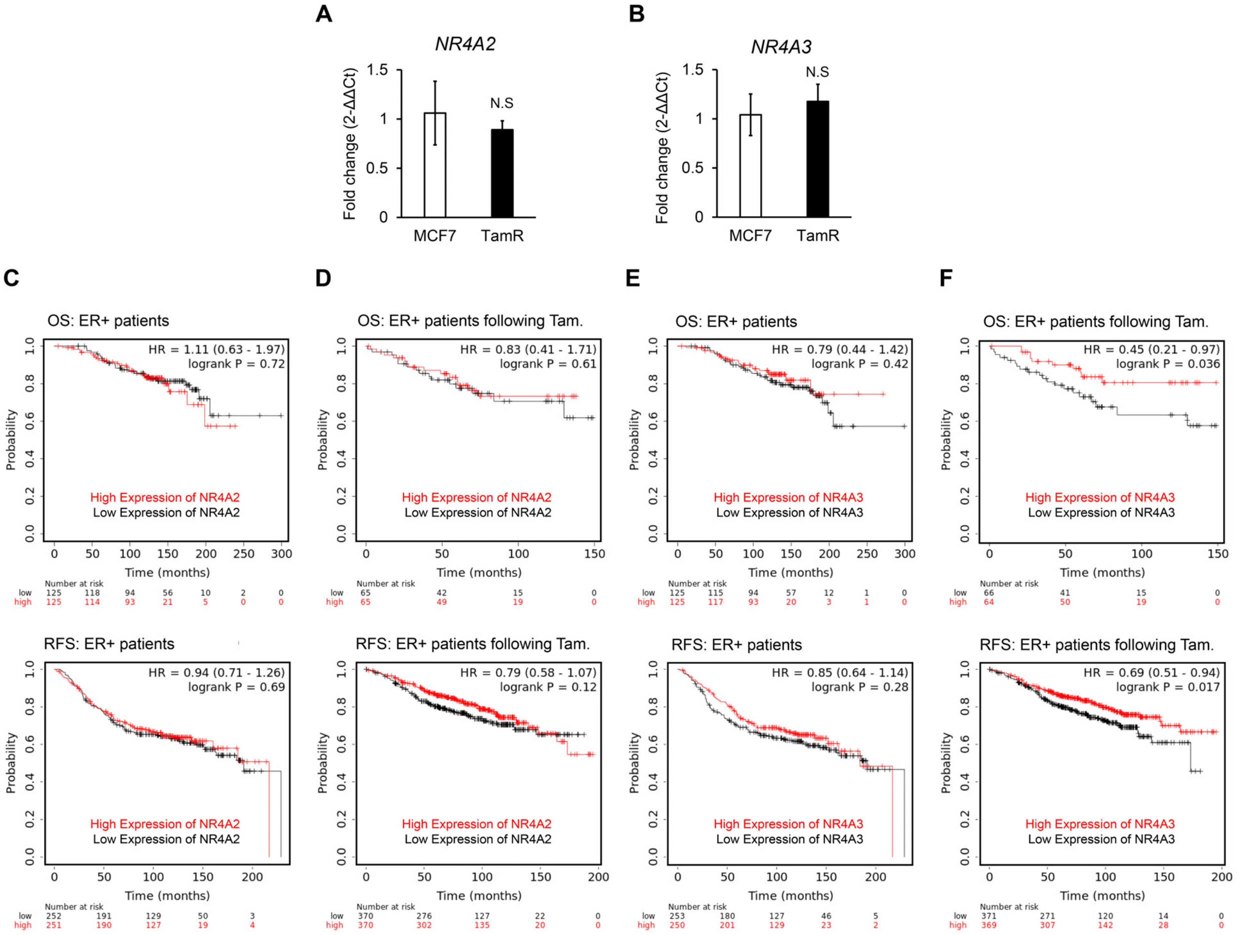

Figure S2. NR4A2 and NR4A3 are not associated with tamoxifen sensitivity in ER-positive breast cancer. (**A**) and (**B**) mRNA expression levels of NR4A2 and NR4A3, respectively, in MCF7 and TamR breast cancer cells. (**C**) Kaplan-Meier analysis curves of overall survival (OS; upper panel) (n = 250, $p = 0.72$) and recurrence-free survival (RFS; lower panel) (n = 503, $p = 0.69$) rates of patients with ER-positive breast cancer receiving no treatment. (**D**) OS (upper panel; n = 130, $p = 0.61$) and RFS (lower panel; n = 503, $p = 0.12$) curves for patients following tamoxifen therapy. The

NR4A2 (204622_x_at) probe was used and analyzed to detect survival rates (**E**) Kaplan-Meier analysis curves of OS (upper panel; n = 250, p = 0.42) and RFS (lower panel; n = 503, p = 0.28) rates of patients with ER-positive breast cancer receiving no treatment. (**F**) OS (upper panel; n = 130, p = 0.036) and RFS (lower panel; n = 503, p = 0.017) curves for patients following tamoxifen therapy. The NR4A3 (209959_at) probe was used and analyzed to detect survival rates. N.S = not significant.

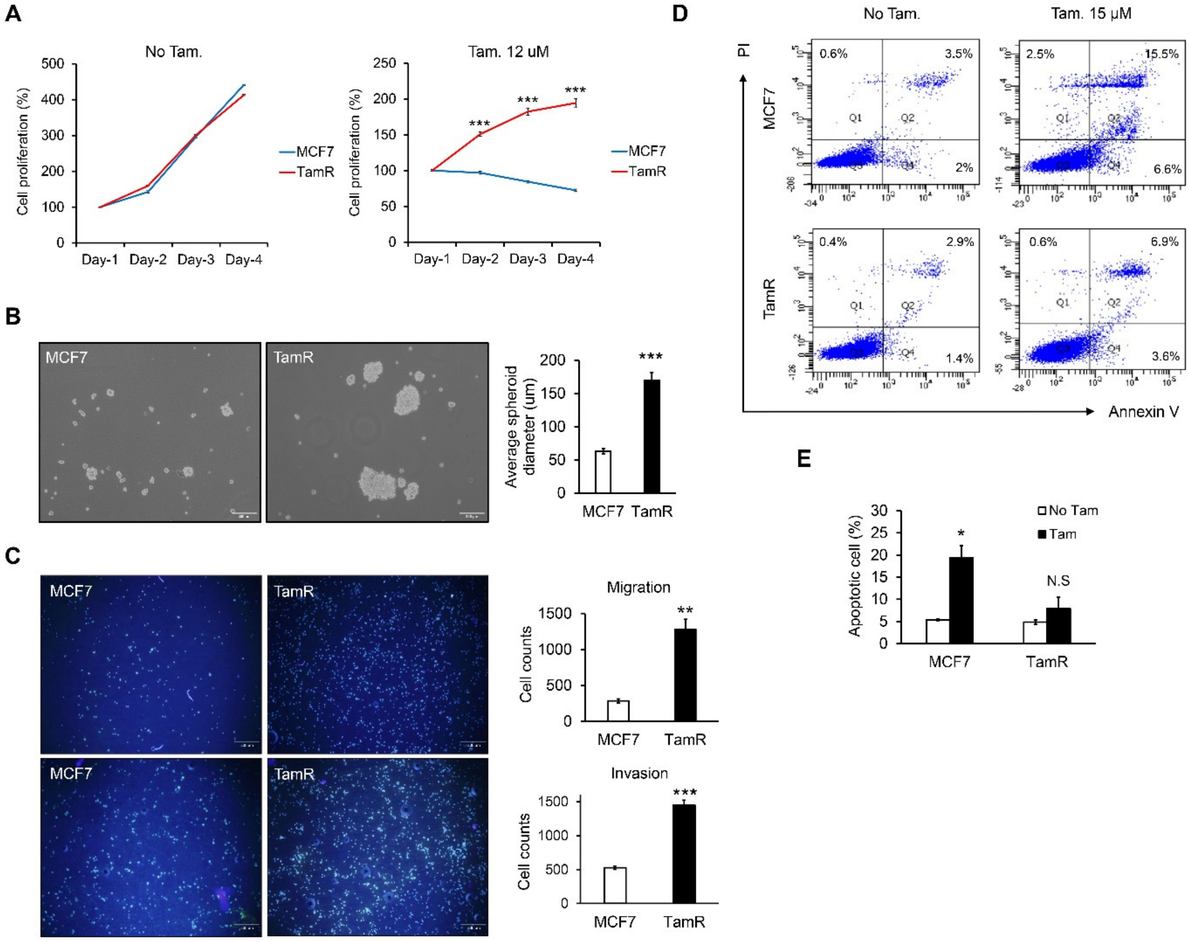

Figure S3. TamR cells are more resistant to tamoxifen compared to MCF7 cells. (**A**) Cell proliferation curves of MCF7 and TamR cells untreated (No Tam.) or treated with 12 µM tamoxifen (Tam.) for 4 days. (**B**) Representative images of breast cancer spheroids growth of MCF7 and TamR cells (left panel). Spheroid formation was analyzed after 6 days. Quantification of average spheroid diameter (right panel) is represented on a bar graph. (**C**) Matrigel invasion and migration assays in MCF7 and TamR cells. Migrating and invasive cells were stained with DAPI. (D) FACS analysis of apoptotic cell death in MCF7 and TamR cells untreated or treated with 15 µM tamoxifen. Apoptotic cells were stained by Annexin V/PI. (E) Percentage of apoptotic cells from (**D**) are represented on a bar graph. * $p < 0.05$, ** $p < 0.01$, and *** $p < 0.001$.

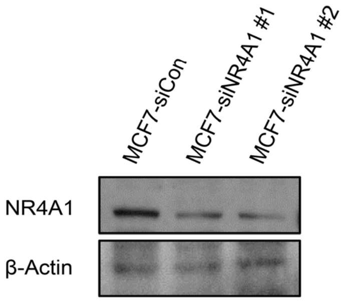

Figure S4. The efficiency of NR4A1 knockdown in MCF7 cells. Western blot analysis of NR4A1 in MCF7-siCon, MCF7-siNR4A1 #1 and #2 cells.

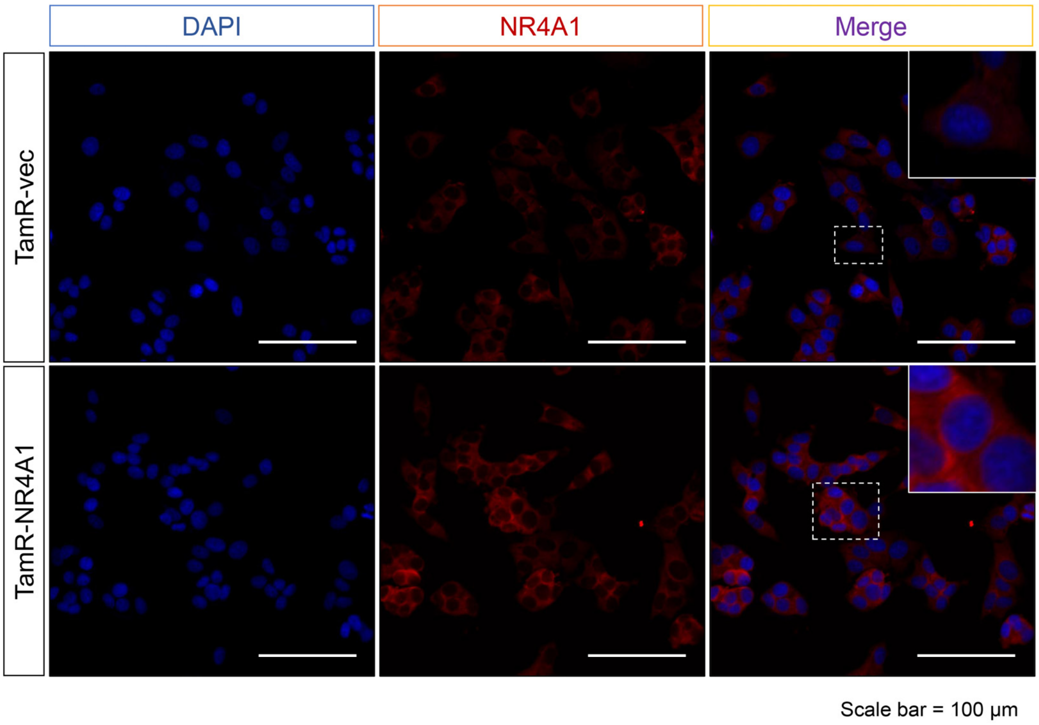

Figure S5. NR4A1 localizes in the cytoplasm following NR4A1 overexpression in TamR cells. Representative images of immunofluorescence for NR4A1 localization in TamR-vec and TamR-NR4A1 cells. Scale bar = 100.

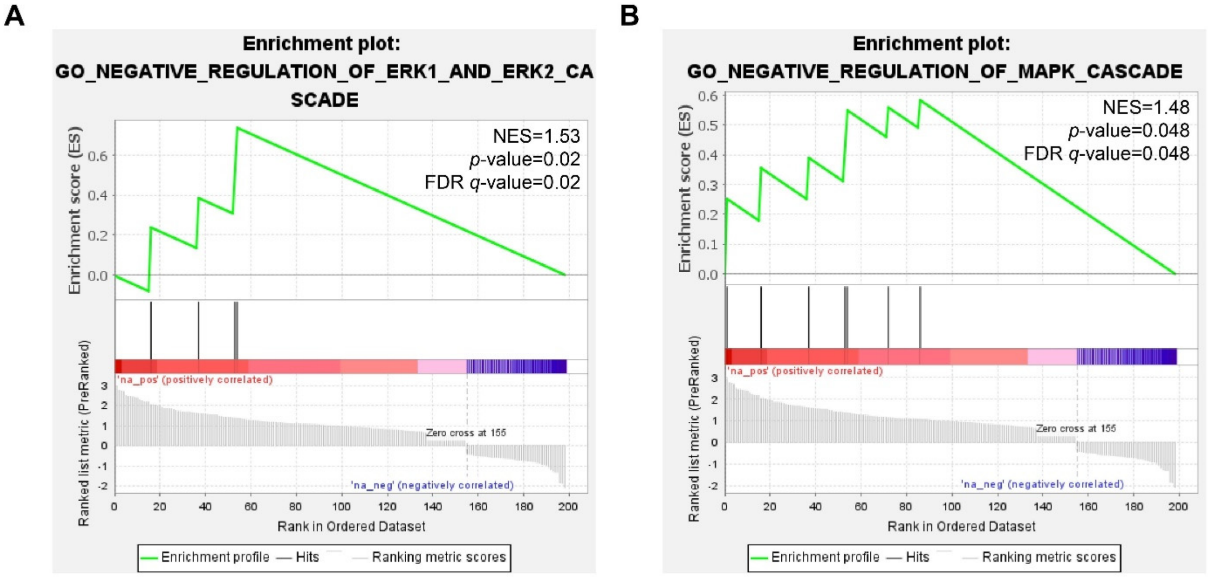

Figure S6. High NR4A1 is related to the negative regulation of ERK1, ERK2, and MAPK signaling pathways.Gene set enrichment analysis plots showing a significant correlation between high mRNA levels of NR4A1 and negative regulation of ERK1, ERK2 **(A)**, and MAPK cascade **(B)** gene sets in a breast cancer patient dataset retrieved from cBioportal. NES, normalized enrichment score; FDR, false discovery rate.
